# Supplementary material for: The interactome of intact mitochondria by cross-linking mass spectrometry provides evidence for coexisting respiratory supercomplexes
Source: Mol Cell Proteomics. 2017 Dec 8;17(2):216–32. doi: 10.1074/mcp.RA117.000470 (PMC5795388; doi:10.1074/mcp.RA117.000470)
Supplement: Supplemental Data [file supp_RA117.000470_133922_0_supp_23978_fzffwf.zip › spectra_annotation/mito_DR_spectra_annotation/235-1-22-1-2-1.pdf]

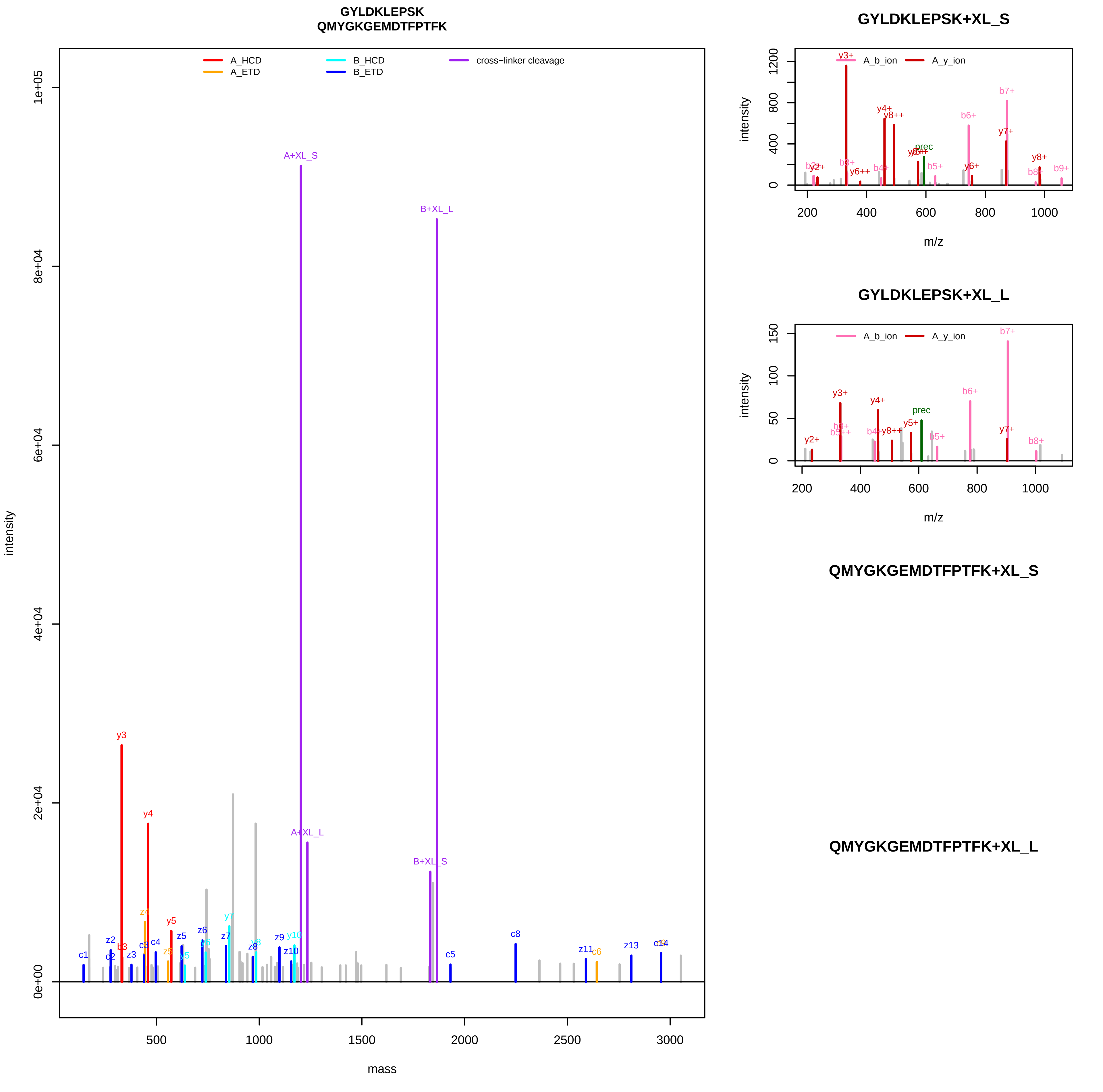
GYLDKLEPSK
QMYGKGEMDTFPTFK
A_HCD
A_ETD
B_HCD
B_ETD
cross−linker cleavage
A+XL_S
B+XL_L
A+XL_L
B+XL_S
y3
y4
y5
z4
z5
c1
c2
z2
b3
z3
c3
c4
z5
z6
y5
y6
z7
y7
z8
y8
z9
y10
z10
c5
c8
z11
c6
z13
c14
intensity
mass
0e+00
2e+04
4e+04
6e+04
8e+04
1e+05
500
1000
1500
2000
2500
3000
GYLDKLEPSK+XL_S
A_b_ion
A_y_ion
y3+
y4+
y8++
b6+
b7+
y7+
prec
y5+
b5+
y6+
y8+
b8+
b9+
y2+
b3+
y6++
b4+
m/z
intensity
0
400
800
1200
200
400
600
800
1000
GYLDKLEPSK+XL_L
A_b_ion
A_y_ion
b7+
b6+
y3+
y4+
prec
y5+
b3+
b5++
b4+
y8++
b5+
y7+
y2+
b8+
m/z
intensity
0
50
100
150
200
400
600
800
1000
QMYGKGEMDTFPTFK+XL_S
QMYGKGEMDTFPTFK+XL_L
